# Some Appreciations and a Suggestion

**Published:** 1913-10-18

**Authors:** 


### HOSPITALS FROM THE PATIENTS' POINT OF VIEW.

(Contributions to this Section are Invited.)

### Some Appreciations and a Suggestion.

I was sent into a general hospital in London by my doctor for a major operation. On my arrival there were the usual preliminaries of bathing, etc., to be gone through. But as I was a private patient I was excused from having my hair combed through with a small dust-comb. This was the case all the time I was in hospital, although the seventeen other patients in the ward were so attended to every day. I soon realised that my exemption was a doubtful privilege, for in long, thick hair an uncomfortable amount of dust quickly accumulates when lying in bed amidst the inevitably large amount of traffic up and down a busy ward. Amongst hospital patients there is an amazingly false pride concerning the use of a dust-comb, and both from the point of view of personal comfort and that of the precaution of general cleanliness in the ward, I should like to see each patient provided with a small dust-comb, which should be used daily.

With regard to "hospital noises," none were serious enough for complaint, except those caused by the presence of three babies in the ward. I consider it is always a great disadvantage to have children in a ward amongst sick adults, a fact which should be obvious to all hospital managers.

In this hospital the middle lights in the wards were hung over pulleys an excellent arrangement which enabled them to be slung over a screen for any necessary treatment during the night, and so doing away with the necessity of turning on a side light and running the risk of disturbing the patient in the next bed. The nurses' work began at 4 A.M. Much of this early disturbing of patients and consequent complaining would be avoided if only nurses had an eight-hour day, and there were a triple shift.

The patients were very happy, and one heard expressions of gratitude on every side. But there was one thing that was a trial to one and all alike—the use of the bed-pan. All patients should be induced to try to use the pan at the regular times when it is brought round. But impossible as it seems for healthy, active people to manage their insides, far more so is it for sick people lying in bed all day. Aperients were given at night. If these did not act during the night, or when there was the opportunity at five in the morning, they often began to make the patients feel very uncomfortable after their hot tea at breakfast time. But there were many times when it was only with the greatest difficulty that the over-tired and over-rushed night nurse could be induced to bring round more bed-pans. And woe betide the patients who made such a request to the day nurses at the beginning of the morning rush. Here again the patient suffers through the overstrain of the nurses.

A word here too with regard to constipation in patients who have recently undergone abdominal operations. Even before my operation I had been for a long time distinctly below par. When, therefore, I was allowed to become very constipated, straining at stool and consequent abdominal pain were not conducive to satisfactory progress. Patients in a voluntary hospital should not expect luxuries, but there are some minor details which would materially help their comfort. For instance, when a patient is first allowed to sit up in bed, the position of having only the two somewhat thin pillows to prop him up is a very tiring one. Expensive bed-rests are not necessary to surmount this difficulty. Firm chaff pillows, with a suitable covering placed behind or beneath the patient's own, will answer admirably. A rest of some sort under the knees also is an invaluable help, especially during the tedious time between being allowed to sit up in bed and really getting up. Although the bed-clothes were excellent, I heard many patients complaining of cold feet. It is not possible or advisable for all patients to be provided with hot-water bottles, but all should come, or be provided, with long bed-stockings to reach over the knee. Short ones are always slipping off, and they are best knitted in one long, straight piece, without heel, as they do not wear so quickly, and later can be turned "top to bottom."

Also with regard to open windows—would it not be better for each patient, men and women, to have a light wrap to throw over their head or shoulders if any inconvenience is felt, rather than the windows should be closed as is so often the case?

In the face of Sir Wm. Osler's remarks at a recent conference, I should like to see each patient on leaving hospital presented with an account of the real value of the treatment he has been able to receive at the hospital's hands—*e.g.* to operation for removal of appendix abscess, so many guineas. To so many ozs. medicine at so much per oz. To massage at so much per hour. To professional nursing at so much per week. And so on. This account could be accompanied by the receipt for the actual payment made by, or for, the patient in question. It is so usual a thing to hear the working-people say they "can't think what becomes of all the money that is given to the hospital."

Such a plan as is mentioned above would give some idea of the difficulties of making ends meet in hospital finances, and of the real value of medical, surgical, and nursing aid which they would have to pay fully for were they placed one or two stages higher in the social scale.

I have seen patients amazed when told the value of a clinical thermometer, which they generally think can be got for threepence, and to their minds all other hospital adjuncts seem to be on the same scale. The plan I have mentioned would need to be very carefully thought out. The idea of placing patients under an obligation must be avoided. But as they are so often subscribers in accordance with their means, through some such agency as the Hospital Sunday and Saturday Funds, etc., they have some right to know something of the details of the expenditure of the hospital funds, and of the real value of the work they try to help on.

